# Role of High-Frequency Oscillation in Locating an Epileptogenic Zone for Radiofrequency Thermocoagulation

**DOI:** 10.3389/fnhum.2021.699556

**Published:** 2021-09-24

**Authors:** Xin Xu, Xingguang Yu, Guixia Kang, Zhiqi Mao, Zhiqiang Cui, Longsheng Pan, Rui Zong, Yuan Tang, Ming Wan, Zhipei Ling

**Affiliations:** ^1^Department of Neurosurgery, General Hospital of PLA, Beijing, China; ^2^Key Laboratory of Universal Wireless Communications, Ministry of Education, Beijing University of Posts and Telecommunications, Beijing, China

**Keywords:** high-frequency oscillation, radiofrequency thermocoagulation, gray matter nodular heterotopia, epilepsy, stereotactic-electroencephalogram

## Abstract

Radiofrequency thermocoagulation (RFTC) has been proposed as a first-line surgical treatment option for patients with drug-resistant focal epilepsy (DRE) that is associated with gray matter nodular heterotopia (GMNH). Excellent results on seizures have been reported following unilateral RFTC performed on ictal high-frequency-discharge, fast-rhythm, and low-voltage initiation areas. Complex cases (GMNH plus other malformations of cortical development) do not have good outcomes with RFTC. Yet, there is little research studying the effect of high-frequency oscillation in locating epileptogenic zones for thermocoagulation on unilateral, DRE with bilateral GMNH. We present a case of DRE with bilateral GMNH, treated using RFTC on unilateral GMNH and the overlying cortex, guided by stereotactic electroencephalogram (SEGG), and followed up for 69 months. Twenty-four-hour EGG recordings, seizure frequency, post-RFTC MRI, and neuropsychological tests were performed once yearly. To date, this patient is seizure-free, the electroencephalogram is normal, neuropsychological problems have not been found, and the trace of RFTC has been clearly identified on MRI. His dosage of antiepileptic medication has, furthermore, been significantly reduced. It is concluded that RFTC on unilateral DRE with bilateral GMNH may achieve good long-term effects, lasting up to, and perhaps longer than, 69 months. Ictal high-frequency oscillation (fast ripple) inside the heterotopia and the overlying cortex may be the key to this successful effect.

## Introduction

Epilepsy is one of the most prevalent neurological disorders, with around 70 million individuals affected worldwide. It can cause severe neurological disorders, leading to significant morbidity and increased mortality. Surgery should be considered in patients with drug-resistant focal epilepsy (DRE) and lesion-based epilepsy. Histopathology is an important determinant of seizure outcome (Lamberink et al., 2020). Gray matter nodular heterotopia (GMNH) often leads to DRE as a result of malformations arising during cortical development (Guerrini and Filippi, 2005; Battaglia et al., 2006). However, the relative roles of the nodular tissue and the overlying cortex in the generation of seizures can be complex and variable (Aghakhani et al., 2005; Tassi et al., 2005; Thompson et al., 2016). Radiofrequency thermocoagulation (RFTC) has been proposed as a surgical treatment option in patients with DRE associated with GMNH (Cossu et al., 2015, 2018), with excellent outcomes reported. Cossu et al. (2014) indicated that four out of five patients experienced sustained seizure freedom for 33.5 months following the coagulation of a single, unilateral GMNH. Mirandola et al. (2017) showed that stereotactic-electroencephalogram (SEEG)-guided RFTC was effective in 15/20 (76%) patients with DRE related to GMNH.

Radiofrequency thermocoagulation was performed in the region of ictal high-frequency, mainly in the fast-rhythm and low-voltage initiation area, guided by SEEG, which is a key factor in achieving a long-term effect (Guenot et al., 2004; Scherer et al., 2005; Cossu et al., 2018). RFTC, especially if guided by SEEG evaluation, should be considered as a first-line treatment option in single, unilateral GMNH-related epilepsy (Bourdillon et al., 2017). Outcomes with complex cases (GMNH plus other malformations of cortical development) are, however, not as good as those in other patterns of GMNH (Cossu et al., 2018). Furthermore, a worse result is obtained with bilateral GMNH, especially when the nodules are asymmetric and likely to be due to the presence of multifocal epilepsy, as a result of which the patients tend to experience multiple seizures (Battaglia et al., 2006; Thompson et al., 2016). There is, therefore, a need to study the role of high-frequency oscillation in locating the epileptogenic zone for RFTC in DRE cases with bilateral GMNH.

In this study, we present a case of DRE in which the seizure frequency was two to three times per month. The main seizure semiology was dizziness → blurred vision, with bilateral GMNH, treated using monolateral SEEG-guided RFTC, and followed up for 69 months. Twenty-four-hour electroencephalography (EEG) recordings identifying seizure frequency, post-RFTC MRI, and neuropsychological tests were performed per year. The patient and his mother provided written informed consent for the details of this case to be used for publication.

## Case Description

**FIGURE 1:**
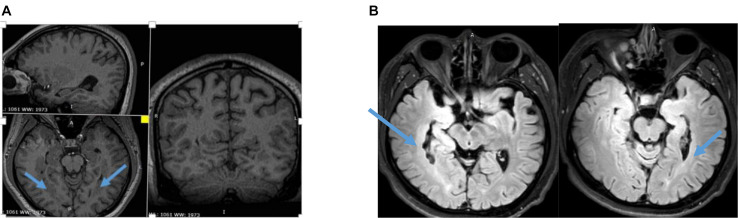
**(A)** Three-dimensional (3D) MRI showing bilateral periventricular heterotopia and overlying polymicrogyria. **(B)** T2 Flair of MRI showing asymmetry bilateral periventricular heterotopia and overlying polymicrogyria.

An 18-year-old male patient with a 4-year history of epilepsy visited the Department of Neurosurgery, General Hospital of PLA, in July 2015. He had undergone normal gestation and development and had no family history of epilepsy, febrile convulsions, encephalitis, or brain trauma. MRI of his brain showed bilateral periventricular heterotopia with overlying polymicrogyria of the bilateral temporal lobes (Figure 1). Neuropsychological and visual field tests were normal. The antiepileptic drug oxcarbazepine (450 mg/300 mg/450 mg) had been administered for 1 year. The seizure frequency was two to three times per month at the time of his examination.

## Case Progression Timeline

This patient experienced a seizure for the first time when he was 14 years old. It occurred one night in the summer of that year when he suddenly turned his head to his left side and was unconscious for 2 min. However, he did not have any tonic-clonic movements during this seizure. He reported that he had an aura before turning, with dizziness and blurred vision. From this time onward, he continued to have similar seizures. At times, he had left arm tonic-clonic movements after his head turned to the left side during a seizure. He did not stop having these seizures even though he tried many different antiepileptic drugs in different doses in the preceding 4 years.

## Diagnostic Assessment, Therapeutic Interventions, and Outcomes

**FIGURE 2:**
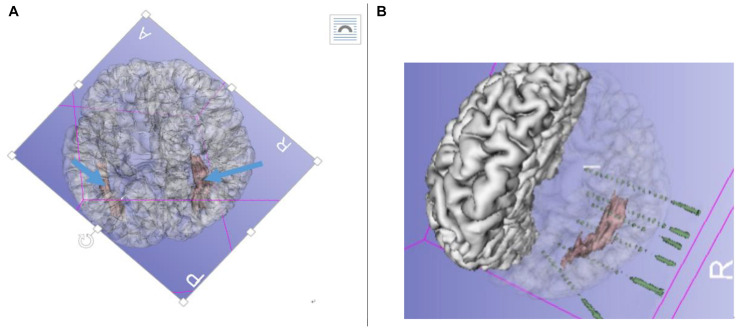
**(A)** Pre-radiofrequency thermocoagulation (RFTC) 3D reconstruction MRI showing bilateral gray matter nodular heterotopia (GMNH), arrow. **(B)** Reconstruction of electrodes superimposed on GMNH.

A preoperative evaluation was performed by two epileptic specialist doctors, and a long-term video scalp electroencephalogram (VEEG) (NicoletOne, Middleton, WI, United States) was administered. The main semiology of the seizures was dizziness → blurred vision → decreased movement (stunned, accompanied by disorders of consciousness) → head deviation to left side → left arm tonic-clonic movements, sometimes followed by general tonic-clonic movements. Ictal discharge shown in the right posterior temporal T6-partial P4-occipital O_2_ area was fast rhythm (ripple) → spike-wave (10/20 international system). Considering the combined MRI results, clinical manifestations, and the epileptic EEG pattern of the patient, we performed implantation using the Robotech-ROSA machine navigation system (Medtech, France). Six electrodes (HuaKe, Beijing, China) were implanted on the right side of the brain. The implantation site included the right temporal lobe, right hippocampus, amygdala, and insula—the anterior, middle, and posterior parts of the heterotopia—and heterotopia in the occipital lobe. No electrodes were implanted in the heterotopic area of the left side because the onset of the seizures and clinical signs were completely on the right side, although there was obvious heterotopia on the left side too (Figure 2).

**FIGURE 3:**
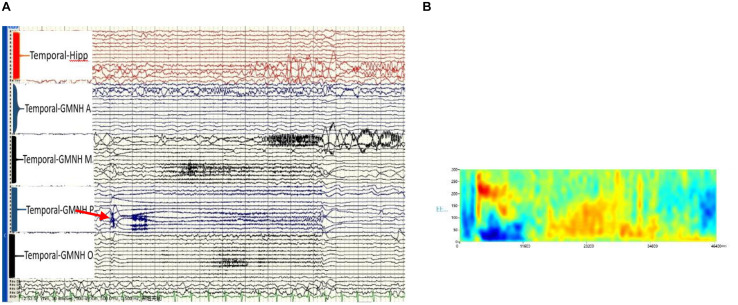
**(A)** Ictal high-frequency oscillation (HFO) of the stereotactic-electroencephalogram (SEEG), post-GMNH showing HFO (ripple), arrow. **(B)** Time frequency showing direct current (DC) shift and HFO, maximum at 300 Hz of the initiation discharge of SEEG. Hipp, hippocampus; GMNH A, anterior part of heterotopia; GMNH M, middle part of heterotopia; GMNH P, posterior part of heterotopia; GMNH O, occipital lobe part of heterotopia.

The patient was transferred to the VEEG room for long-term video monitoring after the implantation. The two epilepsy specialists determined the epilepsy ictal discharge by the SEEG electrodes, which had a total of 74 contacts. Interictal discharges were fast ripples and were only situated at the contacts inside and around the tail end of the heterotopia. The semiology of the seizures was similar to that of the scalp EEG, namely, fast ripple (high-frequency oscillation), maximum 300 Hz, at the initiation of seizure onset, and only situated at the contacts that were the same inside and around the tail end of the heterotopia. Following closely (1 s), the contacts that were within and around the middle of the heterotopia demonstrated a fast rhythm (Figure 3).

Radiofrequency thermocoagulation was used in this patient because of the bilateral GMNH. The patient was not considered to be a suitable candidate for resection. However, high-frequency oscillations (HFOs) were clearly identified in the unilateral heterotopia and the overlying cortex. RFTC was used in those contacts that demonstrated HFOs in interictal and ictal onset, including the posterior parts of the heterotopia and the overlying cortex. Two adjacent contacts were used to perform RFTC (Bei-Qi, China). The stage one pre-RFTC parameters were 2.8 W, temperature of 75°C, continued for 20 s; the stage two sustainability RFTC parameters were 4.8–5.2 W, temperature of 75°C, continued for 30–40 s.

**FIGURE 4:**
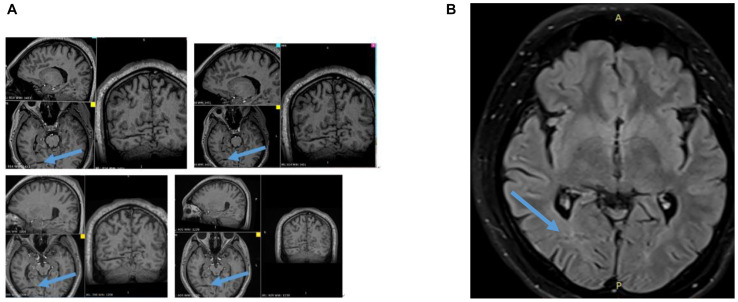
**(A)** Follow-up for 58 months, 3D MRI showing a trace of RFTC at the post-GMNH every year from 2016 to 2019. **(B)** T2 Flair of MRI showing a trace of RFTC at the POST-GMNH and overlying cortex. Blue colored arrow showing a trace of RFTC.

At the time of writing this study, 69 months have passed since the RFTC was performed. The patient has had no seizures since then. Twenty-four-hour EEGs, post-RFTC MRIs, and neuropsychological tests have all been performed once yearly. The EEG results have been normal, and the post-RFTC MRI clearly showed the RFTC site (Figure 4). Neuropsychological tests and visual campimetry test were normal.

Thirty-six months after the RFTC, oxcarbazepine was gradually decreased by one-half tablet every 3 months. At present, the patient is prescribed 150 mg/300 mg of oxcarbazepine and has continued to be seizure-free.

## Discussion

This is a report of a long-term (>69 months) follow-up of a case of DRE with bilateral GMNH and with polymicrogyria within the bilateral temporal region after unilateral RFTC of the GMNH and the overlying cortex guided by SEEG. The patient has been seizure-free for 69 months after the RFTC was performed. Twenty-four-hour VEEG results have been normal, as have neuropsychological tests and a visual campimetry test, despite bilateral GMNH with polymicrogyria within the bilateral temporal lobes.

The SEEG-guided RFTC was key to a successful outcome in this case because SEEG permits the identification of the relationship between nodular heterotopia and the cortex (Stefan et al., 2007). Excellent RFCT results on seizures have been reported following coagulation of only a single, unilateral periventricular nodular heterotopia (Thompson et al., 2016). Heterotopic tissue has been shown to play a leading role in the ictal network in seizures with simultaneous nodular and cortical onset (Dubeau and Tyvaert, 2010; Wu et al., 2012; Agari et al., 2012). However, Mirandola et al. (2017) reported that patients with complex cortical malformations, including GMNH, had less benefit from SEEG-guided RFTC alone. Previous studies have, furthermore, indicated that worse outcomes may occur with multifocal epilepsy, as these patients typically have multiple seizures with bilateral GMNH, especially when the nodules are asymmetric (Cossu et al., 2015). Tassi et al. (2005) reported that ictal onset involves nodules and the overlying cortex. The majority of network seizures (73.4%) arise simultaneously from nodular heterotopia and the overlying cortex (Mirandola et al., 2017).

Unfortunately, not all heterotopia-causing epilepsy information could be obtained from the patient under discussion. However, Acar et al. (2012) also presented a case with unilateral GMNH associated with ipsilateral hippocampal atrophy, but GMNH was not involved in the actual ictal onset zone. The implantation schedule of SEEG in this type of case aims at an intensive sampling of the epileptogenic network for effective RFTC (Cossu et al., 2017). HFOs have been detected in nodular recordings. GMNH may be central to the epileptogenic zone (Jacobs et al., 2009). Wang et al. (2020) suggested that HFOs can be a potential biomarker of epileptogenicity and epileptogenesis due to the strong correlation between HFOs (HFOs > 80 Hz) and the epileptogenic zone.

We present this case of seizures with simultaneous onset in nodular tissue and the overlying cortex with HFO (fast ripple), and a maximum of 300 Hz at the initiation of onset. The patient remained seizure-free for 69 months after RFTC, which was used at the center of the epileptogenic zone, including the heterotopia and the overlying cortex. No treatments, other than RFTC, were provided to these contacts, which demonstrated HFOs at the onset of seizures and included the posterior parts and overlying cortex on only one side of the heterotopia. Our report indicates that not all patients with bilateral GMNH are able to benefit from RFTC. The role of HFOs in locating the epileptogenic zone for RFTC was a very important factor in this case.

## Patient Perspective

Since only a small amount of brain tissue was removed by RFTC, and there was no neuropsychological or visual functional damage, both the patient and his parents were very satisfied with the treatment. They were particularly gratified that he has remained seizure-free, despite a reduction in his dose of antiepileptic medication, and that he takes only 150 mg/300 mg of oxcarbazepine at present.

## Data Availability Statement

The original contributions presented in the study are included in the article/supplementary material, further inquiries can be directed to the corresponding author.

## Ethics Statement

Ethical review and approval was not required for the study on human participants in accordance with the local legislation and institutional requirements. The patients/participants provided their written informed consent to participate in this study. Written informed consent was obtained from the individual(s) for the publication of any potentially identifiable images or data included in this article.

## Author Contributions

XX contributed to the design, construction, and program of the manuscript. ZL contributed to the design and revision of the manuscript. XY contributed to revising the manuscript. GK and YT were involved in data analysis and interpretation. ZM, ZC, and LP contributed to the operation. RZ and MW acquired the data. All authors approved the final version of the manuscript and agreed to be accountable for all aspects of the work and qualify for authorship.

## Conflict of Interest

The authors declare that the research was conducted in the absence of any commercial or financial relationships that could be construed as a potential conflict of interest.

## Publisher’s Note

All claims expressed in this article are solely those of the authors and do not necessarily represent those of their affiliated organizations, or those of the publisher, the editors and the reviewers. Any product that may be evaluated in this article, or claim that may be made by its manufacturer, is not guaranteed or endorsed by the publisher.

## Abbreviations

DRE: drug-resistant focal epilepsy
EEG: electroencephalogram
GMNH: gray matter nodular heterotopia
MRI: magnetic resonance imaging
NH: nodular heterotopia
RFTC: radiofrequency thermocoagulation
SEEG: stereotactic electroencephalogram
VEEG: video scalp electroencephalogram.
